# Tobacco Substitutes: Snus and Harm Reduction

**DOI:** 10.1371/journal.pmed.0040282

**Published:** 2007-10-30

**Authors:** Maia Szalavitz

How can you even have a debate over whether to publish data on snus [1]? No medical journal even contemplated not publishing data on needle exchange to prevent HIV—which continues addiction just as surely as snus does, while similarly producing a dramatic reduction in the risk of death. Journals don't censor data on nicotine replacement or methadone maintenance or buprenorphine or even heroin maintenance, which continue physical dependence if not always addiction. They even publish data on amphetamine maintenance!

What kind of bizarre political correctness would even suggest not publishing data that could show whether snus improves or harms health? You may want to debate whether or not tobacco should be advertised, whether or not the industry should be banned and replaced by government control of nicotine delivery-devices, even whether tobacco should be prohibited entirely. But not publish data on what appears to be from existing data an amazingly successful public health intervention? What kind of a “public library of science” would even contemplate that?
